# Diarrhoeagenic *Escherichia coli *are not a significant cause of diarrhoea in hospitalised children in Kuwait

**DOI:** 10.1186/1471-2180-9-62

**Published:** 2009-03-30

**Authors:** M John Albert, Vincent O Rotimi, Rita Dhar, Susan Silpikurian, Alexander S Pacsa, A Majid Molla, Gyorgy Szucs

**Affiliations:** 1 Department of Microbiology, Faculty of Medicine, Kuwait University, Jabriya, Kuwait; 2 Department of Laboratory Medicine, Farwaniya Hospital, Farwaniya, Kuwait; 3 Department of Paediatrics, Faculty of Medicine, Kuwait University, Jabriya, Kuwait

## Abstract

**Background:**

The importance of diarrhoeagenic *Escherichia coli *(DEC) infections in the Arabian Gulf including Kuwait is not known. The prevalence of DEC (enterotoxigenic [ETEC], enteropathogenic [EPEC], enteroinvasive [EIEC], enterohemorrhagic [EHEC] and enteroaggregative [EAEC]) was studied in 537 children ≤ 5 years old hospitalised with acute diarrhoea and 113 matched controls from two hospitals during 2005–07 by PCR assays using *E. coli *colony pools.

**Results:**

The prevalence of DEC varied from 0.75% for EHEC to 8.4% for EPEC (mostly atypical variety) in diarrhoeal children with no significant differences compared to that in control children (*P *values 0.15 to 1.00). Twenty-seven EPEC isolates studied mostly belonged to non-traditional serotypes and possessed β and θ intimin subtypes. A total of 54 DEC isolates from diarrhoeal children and 4 from controls studied for antimicrobial susceptibility showed resistance for older antimicrobials, ampicillin (0 to 100%), tetracycline (33 to 100%) and trimethoprim (22.2 to 100%); 43.1% of the isolates were multidrug-resistant (resistant to 3 or more agents). Six (10.4%) DEC isolates produced extended spectrum β-lactamases and possessed genetic elements (*bla*_CTX-M_, *bla*_TEM _and IS*Ecp1*) associated with them.

**Conclusion:**

We speculate that the lack of significant association of DEC with diarrhoea in children in Kuwait compared to countries surrounding the Arabian Gulf Region may be attributable to high environmental and food hygiene due to high disposable income in Kuwait.

## Background

Diarrhoeal diseases are a major childhood health problem. Although children in developing countries are the worst affected, children from more developed countries also suffer from diarrhoeal diseases, albeit to a lesser extent. Kuwait is a relatively small country of approximately 17,820 km^2^situated in the desert Arabian Gulf region [1]. It has a population of approximately three million people of which two-thirds are expatriates working for the oil-rich economy [1]. Kuwait is considered a developing country with a high per capita income [2]. The country has a protected piped water supply system. Almost all of the food items are imported from different parts of the world which are routinely screened for microbial safety by the State Public Health Laboratory. Diarrhoeal diseases are a part of the disease spectrum in this country as in other countries. The last study on diarrhoeal diseases in hospitalised children in Kuwait was conducted in early 1980s [3]. That time, not all categories of diarrhoeagenic *Escherichia coli *(DEC) were known. Of late, at least six categories of DEC are known to contribute to disease in different parts of the world. These include enterotoxigenic *E. coli *(ETEC), enteropathogenic *E. coli *(EPEC), enteroinvasive *E. coli *(EIEC), enterohaemorrhagic *E. coli *(EHEC), enteroaggregative *E. coli *(EAEC) and diffusively adherent *E. coli *(DAEC)[4]. However, Koch's postulates have been fulfilled for five categories excluding DAEC [5]. Therefore, we investigated the aetiology of the five categories of DEC in hospitalised children with diarrhoea in Kuwait to assess their importance in this part of the world. DEC isolates were further characterised for their antimicrobial susceptibility and extended spectrum β-lactamase (ESBL) production. In addition, the EPEC isolates were characterised for their serotypes and intimin subtypes [6].

## Methods

### Subjects

The subjects included 537 consecutive children hospitalised with acute diarrhoea (defined as three or more loose stools during a 24 h period with duration of diarrhoea ≤ 14 days) and 113 control children without diarrhoea. The diarrhoeal children were hospitalised because of dehydration. The children were up to five years of age and were recruited from Al-Adan Hospital (AH) or Al-Farwaniya Hospital (FH), Kuwait, during August 2005 to March 2007. Control children were admitted for non-gastrointestinal illnesses, but were matched for corresponding age of the diarrhoeal children. The children had not taken antibiotics prior to hospital admission and there was no follow-up of them after stool sample collection. Informed oral consent was given by the parents or guardians of children for the study as per local institutional guidelines.

### Stool samples

A fresh stool specimen was collected from children with diarrhoea, and from control children without diarrhoea, as soon as after admission. It was promptly sent to the Microbiology Laboratory of each hospital where it was cultured on MacConkey agar (Oxoid, Basingstoke, UK). The plate was incubated at 37°C for 24 h. The next day, the MacConkey plate (Oxoid) and the stool specimen were sent in a refrigerated box to Department of Microbiology, Faculty of Medicine, Kuwait University.

### Detection of DEC

Entire *E. coli *growth from MacConkey plate (including both lactose fermenting and non-lactose fermenting colonies) was transferred to Luria broth (Becton Dickinson, Franklin Lakes, NJ, USA) containing 30% (vol/vol) glycerol, which was then frozen at -70°C until studied for detection of ETEC, EPEC, EIEC, EHEC and EAEC by PCR assays as described by Robins-Browne et al [7]. For detection of these DEC, a loopful of the frozen culture was grown in 2.5 ml of MacConkey broth (Oxoid) in a shaker incubator at 37°C overnight. The pelleted bacterial growth was washed in 1 ml of phosphate buffered saline (PBS)(pH, 7.2), resuspended in 200 μl sterile distilled water, and boiled for 10 min. After cooling on ice, bacteria were pelleted by centrifugation and supernatant stored for ≤ 1 week at -20°C before use. PCR reaction was carried out in a total volume of 25 μl using 5 μl of thawed supernatant diluted 1: 5 in PBS (pH, 7.2) as the template in all PCR reactions. Initially, the presence of *E. coli *was checked by PCR reaction for *lacZ *gene [7]. If positive, then PCR assays for DEC were carried out. The primers and the PCR conditions corresponded to *lac Z *gene [7], *eltA *and *estA *genes (for ETEC), *bfpA *and *eaeA *genes (for EPEC), *stx1*and *stx2 *genes (for EHEC), and *AggA *gene (for EAEC) [7] and *ipH *gene (for EIEC) [8]. The PCR buffer contained 10 mM Tris-HCl (pH 8.3), 25 mM MgCl_2_, 10 mM each of dnTP, and 1 unit of Taq Gold polymerase (Amplitaq gold, Applied Biosystems, Branchburg, NJ, USA) and 6.5 pmol each of the primer. The reaction volume was made up to 25 μl with distilled water. The following *E. coli *control strains were used in PCR reactions: EPEC strain, 2348/69; EHEC strain, EDL 933; ETEC strain, H10407; EIEC strain, 223–83; and EAEC strain, O42 (provided by Professor R. Robins-Browne, University of Melbourne, Parklville, Victoria, Australia). Amplified DNA fragments were resolved by gel electrophoresis with 2% (wt/vol) agarose. The gels were stained with ethidium bromide (0.5 μg/ml) and bands visualised with UV illumination.

### Isolation of DEC from mixed *E. coli *growth

Frozen *E. coli *growth from individuals positive for DEC were replated on MacConkey agar (Oxoid) for isolated colonies and up to 10 individual colonies were tested for the DEC initially identified in the pooled growth. Growth from single colonies identified as DEC was stored frozen at -70°C for further studies on intimin subtyping (EPEC isolates only) and antimicrobial susceptibility (all DEC isolates) (see below).

### Subtyping of the eae gene

The subtyping of intimin from EPEC strains into 14 subtypes was carried out as described by Ramachandran et al [6]. A single forward primer (EaeVF) and three reverse primers (EaeVR, EaeZeataVR and EaeIotaVR) were used to amplify a 834- to-876-bp fragment representing the 3' variable regions of the reported intimin variants. The composition of the PCR buffer was as above, but 50 pmol of each primer was used. The template (5 μl) used was the same as above for identification of EPEC. The reaction volume was made up to 50 μl with distilled water. After amplification, the DNA products were resolved by agarose gel electrophoresis as described above. The PCR products generated with the cocktails of the four primers were incubated separately with 3 U of each of the restriction enzymes *Alu*I, *Rsa*I, and *Cfo*I (New England Biolabs, Ipswich, MA, USA) for 4 h at 37°C. The digested fragments were separated by agarose gel electrophoresis and visualised by ethidium bromide staining. Intimin subtypes were identified by comparing the generated profiles with those reported previously [6]. Any profile that did not fit with the published profiles was considered to be of indeterminate type [6].

### Serotyping of EPEC strains

Selected EPEC isolates from diarrhoeal children and control children were serotyped at the Health Protection Agency's Laboratory of Enteric Pathogens, Colindale, England, the United Kingdom, by tube agglutination method [9].

### Antibiotic susceptibility testing of DEC

DEC strains were tested for susceptibility to a number of antimicrobial agents by E test (AB Biodisk, Solna, Sweden). Bacterial suspension in Mueller-Hinton broth (Difco, Becton Dickinson, NJ, USA) equivalent to 0.5 McFarland optical density was used to inoculate Mueller-Hinton agar. E test antimicrobial strips were placed on the plate, which was then incubated at 37°C for 20 h. The MIC value was read where the growth inhibition ellipse intersected the antibiotic gradient concentration. The susceptibility test was controlled by the quality control organism, *Escherichia coli *ATCC 25922. The test bacteria were categorised as susceptible or resistant as per published criteria [10].

### Detection of genes mediating ESBL production

All DEC strains were screened for ESBL production by the Etest ESBL method using both ceftazidime/ceftazidime combined with clavulanic acid and cefotaxime/cefotaxime combined with clavulanic acid acid strips (AB Biodisk) as described previously [11]. The three common *β*-lactamase-encoding genes, *bla*_TEM_, *bla*_SHV _and *bla*_CTX-M_and the insertion sequence mobilizing the *bla*_CTX-M _gene, IS*Ecp1 *were detected by PCR assays as described previously [11]. The expected amplicon sizes were 971 bp (*bla*_TEM_), 798 bp (*bla*_SHV_), 543 bp (*bla*_CTX-M_) and 527 bp (IS*Ecp*). The PCR products of *bla*_CTX-M _and IS*Ecp *genes were sequenced and compared with the sequences in the public data bank by BLAST (Basic Local Alignment Search Tool) algorithm to determine their types.

### Statistics

The significance of the difference in the prevalence of pathogens between patients and controls was calculated by Chi square test. A *P *value ≤ 0.05 was considered significant.

## Results

The age stratification of children with diarrhoea and control children from AH and FH is shown in Table 1. The majority of the patients and controls were ≤ 2 years of age. The detection of DEC from case-control study of children in AH and FH is shown in Table 2. A total of 85 (15.8%) diarrhoeal children harboured a DEC. Among these 85 children were 2 children with dual infections: 1 had an EPEC and EAEC and the other ETEC and EIEC. The prevalence was greatest for EPEC among patients. Comparison of prevalence of EPEC between patients and controls did not show statistically significant difference. Of the 45 patients positive for EPEC, 21(6.01%) were up to two years of age. Of the 8 control children positive for EPEC, 7 (7.95%) were up to two years of age. There was no significant difference in the prevalence of EPEC between patients and controls up to 2 years of age (*P *= 0.68). Only 2 patients harboured typical EPEC (positive for both attaching and effacing gene and bundle-forming pilus gene). The other 43 patients and all 8 controls positive for EPEC harboured atypical EPEC isolates (positive for attaching and effacing gene only). The other categories of DEC were present in a small number of patients and not in controls.

**Table 1:** Age strata of 537 diarrhoeal children and 113 control children from Al-Adan and Al-Farwaniya hospitals, Kuwait

Age (months) strata	Number of diarrhoeal children	Number of control children
0–12	250	69
13–24	99	19
25–36	88	8
37–48	60	8
49–60	40	9

**Table 2:** Detection of diarrhoeagenic *E. coli *from 537 diarrhoeal children and 113 control children from Al-Adan and Al-Farwaniya hospitals, Kuwait

	No (%) of infected children		*P *value
		
Organism	Diarrhoeal	Control	
ETEC^a^	10 (1.86)	0 (0.00)	0.22
EPEC^b^	45 (8.38)	8 (7.08)	0.85
EIEC^c^	12 (2.24)	0 (0.00)	0.24
EHEC^d^	4 (0.75)	0 (0.00)	1.00
EAEC^e^	14 (2.61)	0 (0.00)	0.15

^a^Enterotoxigenic *E. coli *^b^Enteropathogenic *E. coli*

^c^Enteroinvasive *E. coli *^d ^Enterohaemorrhagic *E. coli*

^e^Enteroaggregative *E. coli*

The children with EIEC or EHEC infection did not have bloody diarrhea.

Entire *E. coli *growth from a total of 45 diarrhoeal children and 8 control children was positive for EPEC. On further testing of individual colonies, EPEC colonies could be recovered from 33 diarrhoeal children and 4 control children. Of the 10 diarrhoeal children from both hospitals initially positive for ETEC, ETEC colonies were recovered from 9 children. Of the 12 diarrhoeal children initially positive for EIEC, EIEC colonies could be recovered from 3 children. Of the 14 diarrhoeal children initially positive for EAEC, EAEC colonies could be recovered from 9 children. None of the 4 children initially positive for EHEC yielded EHEC colonies.

The isolated colonies from the above 54 diarrhoeal children and 4 control children were tested for their susceptibilities to 12 antimicrobial agents. The results are summarised in Table 3. There was no resistance to amikacin and imipenem. Resistance to aztreonam, cefotaxime, chloramphenicol, ciprofloxacin, gentamicin and ticarcillin/clavulanic acid was rare. Resistance was significant to ampicillin, tetracycline and trimethoprim. Detailed analysis showed that 16 DEC isolates were susceptible to all antimicrobial agents; six isolates (9.7%) were resistant to 1 agent, 11 isolates (17.7%) were resistant to 2 agents and 25 isolates (43.1%) were resistant to 3 or more agents; and two EPEC isolates, one ETEC isolate and one EAEC isolates were resistant to 7 antimicrobial agents each.

**Table 3:** Antimicrobial susceptibility of diarrhoeagenic *E. coli *isolated from patients and controls from Al-Adan and Al-Farwaniya hospitals, Kuwait

Organism (n)/antibiotic	MIC (μg/ml)			% Resistant
			
	Range	MIC_50_	MIC_90_	
EPEC ^a^(37)				
Amikacin	0.75 – 3	1.5	1.5	0
Ampicillin	3.0 – >256	4	>256	45.9
Ampicillin/sulbactam	0.023 – 64	3	16	29.7
Aztreonam	0.023 – 24	0.047	0.094	5.4
Cefotaxime	0.047 – >256	0.064	0.094	5.4
Chloramphenicol	0.032 – >256	4	8	8.1
Ciprofloxacin	0.006 – 0.25	0.008	0.125	0
Gentamicin	0.004 – 64	0.38	1	8.1
Imipenem	0.094 – 0.25	0.19	0.19	0
Tetracycline	0.5 – 192	1.5	96	40.5
Tircacillin/clavulanic acid	0.75 – 24	2	12	5.41
Trimethoprim	0.19 – >32	1	>32	43.2
				
ETEC ^b^(9)				
Amikacin	1 – 8	2	2	0
Ampicillin	2 – >256	>256	>256	66.7
Ampicillin/sulbactam	1.5 – 24	4	24	33.3
Aztreonam	0.023 – 32	0.032	0.047	11.1
Cefotaxime	0.047 – >256	0.064	3	11.1
Chloramphenicol	2 – 8	4	8	0
Ciprofloxacin	0.004 – >32	0.012	0.032	11.1
Gentamicin	0.25 – 128	1.5	2	11.1
Imipenem	0.094 – 0.75	0.19	0.5	0
Tetracycline	1 – 96	1.5	96	33.3
Tircacillin/clavulanic acid	1.5 – 12	2	8	0
Trimethoprim	0.19 – >32	0.38	>32	22.2
				
EAEC ^c ^(9)				
Amikacin	1 – 4	2	2	0
Ampicillin	>256 – >256	>256	>256	100
Ampicillin/sulbactam	4 – 16	12	16	55.6
Aztreonam	0.016 – 32	0.094	12	33.3
Cefotaxime	0.032 – >256	0.19	>256	44.4
Chloramphenicol	3 – >256	4	8	11.1
Ciprofloxacin	0.004 – 4	0.008	0.19	11.1
Gentamicin	0.38 – 48	1	32	22.2
Imipenem	0.094 – 0.19	0.19	0.19	0
Tetracycline	1.5 – >256	96	192	88.9
Tircacillin/clavulanic acid	1 – 24	12	12	11.1
Trimethoprim	0.38 – >32	>32	>32	77.8
				
EIEC ^d^(3)				
Amikacin	1.5 – 2	1.5	2	0
Ampicillin	1.5 – 4	3	4	0
Ampicillin/sulbactam	1 – 2	1.5	2	0
Aztreonam	0.032 – 0.064	0.047	0.064	0
Cefotaxime	0.032 – 0.047	0.047	0.047	0
Chloramphenicol	3 – 4	4	4	0
Ciprofloxacin	0.004 – 0.125	0.094	0.125	0
Gentamicin	0.19 – 0.75	0.5	0.75	0
Imipenem	0.19 – 0.19	0.19	0.19	0
Tetracycline	32 – 96	96	96	100
Tircarcillin/clavulanic acid	0.38 – 1.5	1.5	1.5	0
Trimethoprim	>32 – >32	>32	>32	100

^a^Enteropathogenic *E. coli *^b^Enterotoxigenic *E. coli *^c^Enteroaggregative *E. coli *^d^Enteroinvasive *E. coli*

Six of the above 58 DEC strains (10.4%) produced ESBL and all of them were isolated from patients with diarrhoea with none from control children. All six strains were resistant to cefotaxime. The types of related genetic elements carried by these strains are shown in Table 4. The strains belonged to EPEC (atypical), EAEC and ETEC categories of DEC. All strains were positive for *bla*_CTX-M _and none carried *bla*_SHV_. Some strains were positive for *bla*_TEM _or IS*Ecp1*.

**Table 4:** Extended spectrum β-lactamase (ESBL)-related genes carried by ESBL-positive strains of diarrhoeagenic *E. coli *(DEC).

		Positive for gene			
		
Strain no.	Category	*bla*_CTX-M_^d^	*bla*_SHV_	*bla*_TEM_	IS*Ecp1*
62	EAEC^a^	14-b	-	+	+
269	EAEC	28	-	-	-
270	EAEC	28	-	-	-
306	EAEC	28	-	-	+
318	ETEC^b^	28	-	+	+
454	EPEC^c^	28	-	+	+

^a^Enteroaggregative *E. coli *^b^Enterotoxigenic *E. coli*

^c^Enteropathogenic *E. coli*

^d ^Type of *bla*_CTX-M _indicated

EPEC colonies recovered from 24 diarrhoeal children and 3 control children were serotyped. (EPEC isolates from 9 diarrhoeal children and 1 control child were accidentally lost while cleaning a freezer). Their intimin subtypes were also determined. The results are presented in Table 5. There were 8 intimin subtypes and many belonged to β, followed by θ. Intimin from one isolate could not be amplified with the four primers used for subtyping. Isolates from 7 children only belonged to the traditional EPEC serotypes (indicated in bold types) [12].

**Table 5:** Serotypes and intimin subtypes of EPEC isolated from children with diarrhoea and control children in Kuwait

Strain number	Serotype	Intimin subtype	Strain number	Serotype	Intimin subtype
23	O121:H19	Int-ε	222	**O111ac:HNT**	Int-ν
24	O118: H8	Int-θ	224	OE8686–77:H21	Int-β
49	O129:H19	Int-ε			
87	ONT: HNT^a^	Int-ξ	229	**O111ac:H-**	Int-β
89	O113:H19	Int-ν	231	ONT:HNT	Int-θ
121	ONT: H2	Int-ε	246	**O26:H-**	Int-β
127	**O55:H-^b^**	Int-θ	251	**O26:H-**	Int-θ
130	O157:H-	Int-κ	252	**O26:H-**	Int-β
151	O123:HNT	Int-ε	257	O85:H31	Int-ζ
153	ONT: H-	Int-θ	319	O98:H-	Int-ζ
159	O145:HNT	Int-α1	341	O85:H31	Int-ζ
174	O85:H-	Int-ζ	C34^d^	ONT:HNT	Int-θ
197	O80:H-	NT^c^	C90^d^	ONT:H7	Int-β
213	O118:H11	Int-β	C87^d^	**O26:H-**	Int-β

^a ^OHT:HNT = O & H antigens non-typeable

^b^Serotypes in bold-face are traditional EPEC serotypes

^c^NT = Intimin non-typeable

^d^Isolates from control children; all other isolates were from children with diarrhoea

## Discussion

All five categories of DEC were present in Kuwaiti children with diarrhoea. The prevalence was greatest for EPEC. However, comparison of its prevalence with control children did not show any significant association of EPEC with diarrhoea. It is believed that EPEC would be associated with diarrhoea in children up to two years of age only [13]. Comparison of prevalence of EPEC in children up to two years of age also did not show significant difference between patients and controls. Other categories of DEC were present only in a small number of patients; none of the controls harboured these organisms. *E. coli *colony pools from some children were initially positive for a DEC. But a DEC could not be detected on subsequent testing of individual colonies. It is likely that DEC were present in very small numbers in these cases that were not detected on screening of individual colonies. Thus, PCR screening of entire bacterial growth from a plate is superior to other methods of detection of pathogens when the pathogens are swamped by normal flora.

Thus, this case-control study suggested that DEC are not epidemiologically associated with Kuwaiti children hospitalised for diarrhoea. Nevertheless, these organisms could still cause diarrhoea in some individual patients. In the previous study conducted in children in Kuwait, the prevalence of ETEC was 9% and EPEC 7% [3]. Compared to that study, the prevalence of ETEC was lower and that of EPEC was similar in the current study. In studies of childhood diarrhoea from the surrounding region, varying prevalence for DEC was observed. In children in Egypt, ETEC contributed to a heavy burden of diarrhoea accounting for 1.5 episodes per child per year [14]. In a study conducted in Tehran, Iran [15], the prevalence of different categories of DEC varied from 7.3% to 44.5% in diarrhoeal cases. In a case-control study of diarrhoea in Tunisian children [16], both cases and controls had a high prevalence of DEC (up to 37%) making an association with diarrhoea difficult. In Bedouin infants in Southern Israel, the prevalence of various categories of DEC varied from 0.2% to 25.9%, but ETEC was the only pathotype significantly associated with diarrhoea [17].

EPEC are classified into two types. Type I or typical EPEC are positive for both *eae *gene and *bfp *gene and mostly belong to the traditional serotypes. Type II or atypical EPEC are positive for *eae *gene only and belong to non-traditional serotypes [18]. In several recent studies [7,19-24], the prevalence of atypical EPEC seems to be on the rise. It is now considered to be an emerging pathogen. In some studies, atypical EPEC was found to be associated with persistent diarrhoea [19,24,25]. Similarly, in our study too, most of the EPEC were of atypical variety and were of non-traditional serotypes. A future study in Kuwait should address whether atypical EPEC are associated with persistent diarrhoea.

The majority of children in our study had nonbloody diarrhoea. Even those children who had EIEC or EHEC detected in their stools, did not present with bloody diarrhoea. It has been reported that in some cases, these infections do not result in bloody diarrhoea [26].

Intimin is the outer membrane protein of EPEC that mediates tight attachment between the bacterium and the intestinal mucosa. We investigated the intimin subtypes of EPEC. There were eight subtypes and the most prevalent subtypes were β and θ. These were also the most frequently identified subtypes in other studies [6,7,24].

Antimicrobial susceptibility studies of DEC showed that resistance to older antimicrobials such as ampicillin, tetracycline and trimethoprim was appreciable and that multi-resistance (resistance to ≥ 3 antimicrobials) was present in 43.1% of the isolates. The resistance rates of DEC to different antimicrobial agents have varied in different studies. In the study in Tehran, Iran, a high prevalence of resistance to above three antimicrobial agents as in Kuwait was observed [15]. In the study in Tunis, Tunisia, a high prevalence of resistance to tetracycline and β-lactams was seen [16]. In ETEC isolates studied in Egypt, a high prevalence of resistance to ampicillin, trimethoprim and tetracycline was seen; 28% of isolates showed multi-resistance; and resistance to other antimicrobials was rare [27]. In Mexico, resistance rates to ampicillin, tetracycline and trimethoprim were high and multi-resistance was 62%; there was no resistance to ciprofloxacin and cefotaxime [28]. In Vietnam, resistance rates to ampicillin, trimethoprim and chloramphenicol exceeded 75% with 90% of all strains multi-resistant. Resistance to ciprofloxacin and imipenem was negligible [29]. A total of six *E. coli *isolates were resistant to a third-generation cephalosporin, cefotaxime. All of them were ESBL producers and possessed one or more genetic elements related to ESBL production. Five isolates had IS*Ecp1 *element that is responsible for mobilization of *bla *genes [30]. There are very few reports of ESBL production by DEC [31-33]. DEC isolates in these studies were found to harbor *bla*CTX-M [31-33], *bla*TEM [32,33] or *bla*PER genes [33]. In Kuwait, children with invasive diarrhea are normally treated with third generation cephalosporins. It is interesting that some of our DEC isolates were resistant to cefotaxime. Therefore, the prevalence of resistance to third generation cephalosporins should be continuously monitored to detect any increase in resistance rate that could affect treatment with this class of antibiotics.

Our study has shown that all five categories of DEC reported from other parts of the world were also present in diarrhoeal children in Kuwait. However, the prevalence of four DEC categories (ETEC, EHEC, EIEC and EAEC) was very low. Even though EPEC was present in about 8% of children with diarrhoea, its prevalence in control children was similar. Thus, the overall burden of diarrhoeal disease due to DEC in Kuwaiti children appeared to be low. This is in contrast to the high burden of diseases due to DEC in countries surrounding the Arabian Gulf Region. We speculate that a number of factors might influence this low prevalence in Kuwait. These include a protected water supply, an arid climate, inspection of imported food items to prevent contaminated food items reaching the population, and better housing, sanitation and nutrition of population because of high disposable income.

There are some limitations in our study. We have studied only severe cases of diarrhoea that required hospitalisation. Therefore, the role of diarrhoeagenic *E. coli *in mild diarrhoeas could not be ascertained. Ideally, we should have studied equal numbers of cases and matched controls. We were able to recruit only a small number of control children because we found it difficult to persuade guardians of children to allow us to collect stool samples from children. Even with a comparatively small number of control children, we could not find a statistical association of DEC with diarrhea as many of these control children excreted DEC. Therefore, even with a larger sample size of control children, the conclusion would have been the same. In most of the diarrhoeal children, other pathogens would have been the cause of diarrhoea. Traditional bacterial and parasitic diarrhoeal pathogens are investigated by routine diagnostic laboratories in the two hospitals on a need basis, but not systematically. Our interest was to evaluate the aetiolo gical role of DEC only. Had we found a significant role for DEC, this would have necessitated ruling out the contribution of copathogens. To our knowledge, ours is the first report of the aetiological role of DEC from the Arabian Gulf region.

## Conclusion

This case-control study has shown that DEC are not significantly associated with acute diarrhoea in hospitalised children in Kuwait.

## Authors' contributions

MJA, VOR, ASP and GS conceived the study and MJA wrote the paper. RD and AMM participated in clinical aspects of the study and specimen collection. SS performed the laboratory studies. All authors read and approved the final manuscript.
